# Antibody-drug conjugates in clinical trials for lymphoid malignancies and multiple myeloma

**DOI:** 10.1186/s13045-019-0786-6

**Published:** 2019-09-10

**Authors:** Bo Yu, Delong Liu

**Affiliations:** 1 Department of Medicine, Lincoln Medical Center, Bronx, NY USA; 2 Department of Oncology, The First affiliated Hospital of Zhengzhou University, Zhengzhou, China; 3 Department of Medicine, New York Medical College and Westchester Medical Center, Valhalla, NY USA

**Keywords:** Antibody-drug conjugate, B cell maturation antigen, Brentuximab vedotin, Inotuzumab ozogamicin, Polatuzumab vedotin

## Abstract

Antibody-drug conjugates (ADC) represent a distinct family of chemoimmunotherapy agents. ADCs are composed of monoclonal antibodies conjugated to cytotoxic payloads via specialized chemical linkers. ADCs therefore combine the immune therapy with targeted chemotherapy. Due to the distinct biomarkers associated with lymphocytes and plasma cells, ADCs have emerged as a promising treatment option for lymphoid malignancies and multiple myeloma. Several ADCs have been approved for clinical applications: brentuximab vedotin, inotuzumab ozogamicin, moxetumomab pasudotox, and polatuzumab vedotin. More novel ADCs are under clinical development. In this article, we summarized the general principles for ADC design, and updated novel ADCs under various stages of clinical trials for lymphoid malignancies and multiple myeloma.

## Introduction

**Fig. 1:**
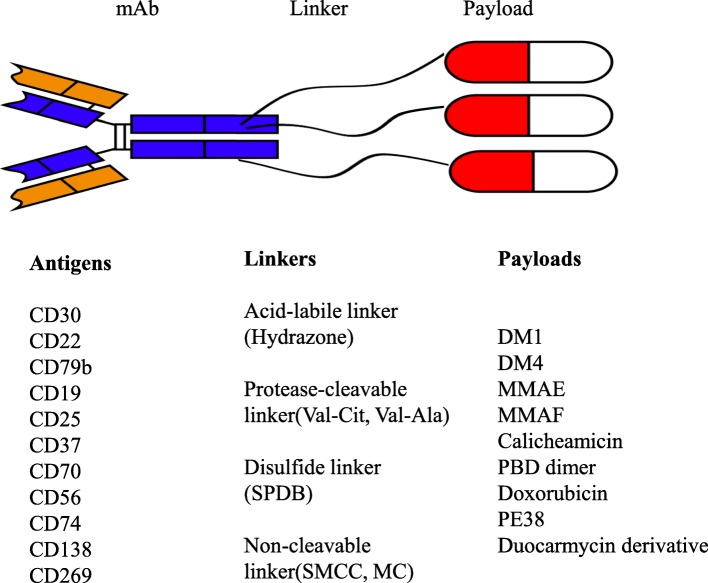
The schematic diagram of the structure of an antibody-drug conjugate. Antigens for the monoclonal antibody (mAb), linker types, and payloads that are in clinical development were listed

Monoclonal antibodies such as rituximab and obinutuzumab are a major component in the combination regimens for the therapy of lymphoid malignancies [1–6]. Antibody-drug conjugates (ADC) are a new class of agents in the treatment of various malignancies. ADC consists of three fundamental elements: a tumor-specific monoclonal antibody (mAb), a cytotoxic small molecule referred to as payload, and a specialized chemical linker that connects the mAb and payload (Fig. 1). Upon binding to the corresponding antigen on the surface of tumor cells, the ADC/antigen complex is internalized and then the payloads are released, leading to cytotoxicity and cell death. ADC represents a novel class of anticancer agents that theoretically enhance targeted killing of tumors while sparing normal tissues, thereby maximizing efficacy and minimizing systemic toxicity [7].

Brentuximab vedotin, inotuzumab ozogamicin, moxetumomab pasudotox, and polatuzumab vedotin are FDA-approved ADCs for lymphoid malignancies [8–11]. More ADCs are under clinical development over the past decade. In this review, we discussed the general principles of ADC design and updated on novel ADCs in clinical trials for the treatment of lymphoid malignancies and multiple myeloma.

## Engineering ADCs

### Selection of antigens and antibodies

An ideal antigen for targeted therapy should have a high copy number on tumor cells with limited or no expression in normal tissues to minimize off-target ADC uptake [12]. The antigen should be able to trigger intracellular internalization upon ADC binding. When an antigen target has heterogenous expression, optimal antitumor activity relies more on the bystander effect, which is referred to as the ADC’s ability to diffuse across cell membranes and exert cytotoxicity on the neighboring cells. This bystander effect is frequently influenced by the chemical nature of the payloads and linkers of the ADCs [13, 14].

Immunoglobulin G (IgG) is the most frequently used subtype in ADCs due to the longer half-life, and the most chosen isotype is IgG1 [15]. IgG1 can induce stronger antibody-dependent cell-mediated cytotoxicity (ADCC) and complement-dependent cytotoxicity (CDC), which further enhance the antitumor effect of ADCs [16]. However, the ADCC and CDC activities intrinsic to the antibodies may add additional toxicities to the cytotoxic payloads. One solution is to engineer the Fc portion of IgG1 heavy chain by introducing mutations to silent the intrinsic effector immune function [17]. IgG2 is believed to conjugate more payloads because it contains four reducible interchain disulfide bonds, whereas IgG1 and IgG4 have only two such bonds [18]. IgG4 has the tendency to exchange with other antibodies, therefore IgG4-based ADCs, such as inotuzumab ozogamicin, often contain a stabilizing mutation in the hinge region to prevent half antibody exchange [19].

### Characteristics of payloads

The payloads used in ADCs are selected small molecules with high potency and proper hydropholicity [20, 21]. Another important parameter is drug-antibody ratio (DAR), which is defined as the average number of payload molecules attached to a single mAb. The ideal level of DAR is between 3 and 4. The DAR affects the drug stability in the circulation, tumor penetration capability, antitumor efficacy, and toxicity of an ADC [22].

The payloads commonly used in ADCs can be divided into two main categories: microtubule inhibitors and DNA-damaging agents. Two currently employed microtubule inhibitors are maytansinoids and auristatins. Maytansinoids were initially derived from maytansine, a natural benzoansamacrolide discovered in the plant *maytenus ovatus* [23]. There are two maytansinoids derivatives: DM1 and DM4. DM1 includes emtansine and mertansine. DM4 includes soravtansine and ravtansine. Auristatins are extracted from the sea hare *Dolabella auricularia*. Two auristatin derivatives are commonly used for ADC constructions: monomethyl auristatin E (MMAE, vedotin) and monomethyl auristatin F (MMAF, mafodotin) [24–31]. MMAE is toxic to the neighboring cells through the bystander effect due to its neutral charge that allows diffusion across cell membranes. MMAE has been used in brentuximab vedotin and polatuzumab vedotin. MMAF lacks the ability of bystander killing [32]. DNA-damaging agents include calicheamicin, pyrrolobenzodiazepines (PBD) dimer, indolinobenzodiazepines, duocarmycins, doxorubicin, etc. [33]. Calicheamicin has been used in inotuzumab ozogamicin and gemtuzumab ozogamycin [11, 34–36].

### Linker selections and conjugation strategies

An ideal linker should not allow premature deconjugation in the circulation which triggers off-target toxicity. Linkers currently used in ADCs fall into two broad categories: cleavable and non-cleavable linkers. Cleavable linkers are sensitive to several intracellular conditions. Here are some examples. Hydrazone, an acid-labile linker used in inotuzumab ozogamicin, can be selectively hydrolyzed in the acidic pH environment inside the lysosomes and endosomes. However, slow hydrolysis under physiologic condition in circulation has been reported [37]. Protease-cleavable linkers contain dipeptide sequences like valine-citrulline (Val-Cit) and valine-alanine (Val-Ala) that can be recognized by cathepsin B. These linkers are often coupled with *p*-aminobenzyloxycarbonyl (PABC) which serves as a spacer between the dipeptide and payload. Protease cleavable linkers show relatively higher stability in plasma. Val-Cit linker has been used to construct brentuximab vedotin [38]. Typical disulfide linkers include *N*-succinimidyl-4-(2-pyridylthio) butanoate (SPDB) and *N*-succinimidyl-4-(2-pyridyldithio) pentanoate (SPP) [39].

Non-cleavable linkers are more stable but rely on complete proteolytic degradation of the whole mAb backbone by the lysosomes to release active payloads. Most common examples of non-cleavable linkers are thioether linkers, *N*-succinimidyl-4-(*N*-maleimidomethyl)cyclohexane-1-carboxylate SMCC) and maleimidocaproyl (MC) [40].

Multiple conjugation strategies have been developed to attach linkers to a specific amino acid residue on the mAb. Lysine is one of the most common amino acid residues used to connect linkers with activated carboxylic acid esters [41]. Cysteine-based conjugation has been used in brentuximab vedotin and a variety of ADCs under development. It has been further improved by a new engineered cysteine technology, THIOMAB, that generates highly homogeneous ADCs with a controlled DAR of 2 [42].

Homogeneous ADCs can also be achieved through site-specific conjugation by incorporating genetically engineered non-natural amino acid (nnAA) [43, 44]. Other conjugation methods include enzymatic conjugations such as transpeptidation mediated by sortase or bacterial transglutaminases, *N*-glycan engineering by β-1,4-galactosyltransferase and α-2,6-sialyltransferase, etc. [45–47].

## ADCs approved for lymphoid malignancies

### Brentuximab vedotin (Adcetris®, SGN-35)

Brentuximab (BV) is composed of an anti-CD30 chimeric IgG1 mAb conjugated to MMAE via a protease-cleavable linker [48]. CD30 is a tumor necrosis factor (TNF) receptor superfamily member, characteristically expressed on the surface of Reed–Sternberg cells in Hodgkin lymphoma (HL) [49], anaplastic large cell lymphoma (ALCL) cells, and a subset of cutaneous T cell lymphoma (CTCL) cells, with limited expression on normal cells [50].

Single-agent BV has been approved by the US FDA in 2011 for the treatment of HL after failure of autologous stem cell transplantation (ASCT) or after failure of at least two prior multiagent chemotherapy regimens [8, 49, 51, 52] (Table 1). In a pivotal phase II trial (NCT00848926), 102 patients with relapsed/refractory (R/R) HL who failed ASCT were treated with intravenous BV 1.8 mg/kg every 3 weeks at a maximum of 16 cycles in the absence of disease progression or unacceptable toxicity. The overall response rate (ORR) was 75% with 34% complete remission (CR) and the median duration of response (DOR) was 6.7 months. The most common treatment-emerging adverse events (TEAE) were peripheral sensory neuropathy (42%), nausea (35%), fatigue (34%), and neutropenia (19%). Fifty-five percent of patients experienced grade ≥ 3 AEs (SAE), majority of which included neutropenia (20%) and peripheral sensory neuropathy (8%). Most of these AEs are manageable with dose reductions and/or delays [8, 53]. At the 5-year follow-up, the overall survival (OS) and progression-free survival (PFS) rate for all patients were 41% and 22% respectively. The estimated median OS and PFS were 40.5 and 9.3 months respectively, suggesting long-term disease control provided by single-agent BV [54]. However, many patients still eventually developed resistance partially because of the upregulation of multidrug resistance pump (MDR1), a drug export pump. Currently, a phase I trial (NCT03013933) is investigating the combination of BV and cyclosporine (CsA), an MDR1 inhibitor, for R/R HL. The interim result was encouraging, showing a 67% ORR (33 % CR) and a manageable toxicity profile at the maximum tolerated dose (MTD) [55].

**Table 1:** FDA approved antibody-drug conjugates for B cell malignancies and multiple myeloma

ADC names	Target	Indications	Dosage and schedule	Year of approval
Brentuximab vedotin (Adcetrix®)	CD30	R/R HL	- 1.8 mg/kg (maximum 180 mg) - Every 3 weeks until disease progression or unacceptable toxicity	2011
		Frontline stage III & IV HL (+ AVD)	- 1.2 mg/kg (maximum 120 mg) combined with chemotherapy - Every 2 weeks until a maximum of 12 doses, disease progression, or unacceptable toxicity	2018
		Post-ASCT consolidation for HL	- 1.8 mg/kg (maximum 180 mg) - Initiate within 4–6 weeks post-ASCT or upon recovery from ASCT - Every 3 weeks until a maximum of 16 cycles, disease progression, or unacceptable toxicity	2015
		R/R systemic ALCL	- 1.8 mg/kg (maximum 180 mg) - Every 3 weeks until disease progression or unacceptable toxicity	2011
		R/R PTCL (+ CHP)	- 1.8 mg/kg (maximum 180 mg) combined with chemotherapy - Every 3 weeks with each cycle of chemotherapy for 6 to 8 doses	2018
		R/R CTCL	- 1.8 mg/kg (maximum 180 mg) - Every 3 weeks until a maximum of 16 cycles, disease progression, or unacceptable toxicity	2017
Inotuzumab ozogamicin (Besponsa®)	CD22	R/R B-cell ALL	- Cycle 1 (21 day-cycle): 1.8 mg/m^2^ [day 1 (0.8 mg/m^2^), day 8 (0.5 mg/m^2^), and day 15 (0.5 mg/m^2^)] - Subsequent cycles (28 day-cycle): 1) Patients who have achieved CR or CRi: 1.5 mg/m^2^ [day 1 (0.5 mg/m^2^), day 8 (0.5 mg/m^2^), and day 15 (0.5 mg/m^2^)] per cycle 2) Patients who have not achieved CR or CRi: repeat cycle 1 - Duration: 1) Patients proceeding to ASCT: 2 cycles 2) Patients not proceeding to ASCT: maximum 6 cycles	2017
Moxetumomab pasudotox (Lumoxiti®)	CD22	R/R HCL	- 0.04 mg/kg on days 1, 3, and 5 of each 28-day cycle. - Maximum of 6 cycles, disease progression, or unacceptable toxicity	2018
Polatuzumab vedotin (Polivy®)	CD79b	R/R DLBCL (+ BR)	- 1.8 mg/kg per cycle, combined with BR - every 21 days for 6 cycles	2019

*ALCL* anaplastic large cell lymphoma, *ALL* acute lymphoblastic leukemia, *ASCT* autologous stem cell transplant, *BR* bendamustine and rituximab, *CR* complete remission, *CTCL* cutaneous T cell lymphoma, *DLBCL* diffuse large cell lymphoma, *HCL* hairy cell leukemia, *HL* Hodgkin lymphoma, *NHL* non-Hodgkin lymphoma, *PTCL* peripheral T cell lymphoma, *R*/*R* relapsed/refractory

BV plus bendamustine (BVB) has been studied as a salvage regimen for R/R HL with favorable toxicity profile in phase I/II trials. One study (NCT01657331) revealed that BVB achieved a 78% ORR in heavily pretreated HL patients. Grade 3 to 4 neutropenia were found in 25% of patients across the trial [56]. Another study (NCT01874054) used BVB as the first salvage regimen for 55 HL patients who failed the frontline therapy. A better outcome was reported with a 92.5% ORR (73.6% CR) and a 62.6% estimated 2-year PFS. Further, 75.4% patients proceeded to ASCT. Most frequently reported SAEs included rash (16.3%), lymphopenia (10.9%), and hypotension (7.3%) [57]. There is another ongoing trial (NCT01657331) that identified 23 out of 65 patients treated with BVB who experienced prolonged median PFS of more than 1 year [58]. BV in combination with nivolumab (Nivo) represents another ongoing clinical trial for R/R HL. In the phase I/II trial (NCT02572167), BV + Nivo achieved 82% ORR and 61% CR, almost doubled the CR rate of BV monotherapy in the pivotal phase II trial. There were mostly grade 1 and 2 AEs: nausea (49%), fatigue (41%), infusion-related reactions (44%) [59]. Currently, BVB and BV + Nivo are being compared in an ongoing phase II trial (NCT02927769).

BV is an effective option of consolidation therapy both before and after ASCT for HL at a high risk of relapse or progression. A randomized, double-blind, multinational, phase III trial (AETHERA, NCT01100502) enrolled 329 eligible patients to receive either 16 cycles of 1.8 mg/kg intravenous BV or placebo every 3 weeks, starting 30–45 days after transplantation. Median PFS was 42.9 months for patients in the BV group (*n* = 165), significantly better than the 24.1 months in the placebo group (*n* = 164) [60]. There was continued benefit at 5-year follow-up, with PFS rate for patients receiving BV and placebo at 59% and 41% respectively [61]. The toxicity profile mainly consisted of peripheral neuropathy (56%) and neutropenia (35%), with neutropenia (29%) being the most common SAE [60]. Recently, a retrospective multicenter study revealed that chemo-refractory HL patients receiving BV prior to allogeneic SCT (AlloSCT) presented with a better outcome and a lower incidence of chronic graft versus host disease (GVHD) at 3-year follow-up compared to those without BV (PFS 53% vs. 33%; OS 62% vs. 44%; GVHD incidence 43% vs. 47%) [62].

BV in combination with chemotherapy has been reported to optimize the frontline treatment of newly diagnosed advanced stage HL. This report was from the international randomized phase III trial (ECHELON-1, NCT01712490) which assigned patients with previously untreated stage III or IV classic HL to receive either BV (adcetris) plus doxorubicin, vinblastine, and dacarbazine (AAVD) (*n* = 664) or doxorubicin, bleomycin, vinblastine, and dacarbazine (ABVD) (*n* = 670) [51]. The outcome of the AAVD cohort appeared to be superior to the ABVD cohort in terms of the 2-year modified PFS (81.0% vs. 74.4%), which was further confirmed by sensitivity analysis, even though the response rate was not significantly different between the two cohorts: ORR (86% vs. 83%), CR (73% vs. 70%). Of note, patients receiving AAVD presented with a higher incidence of peripheral neuropathy (29% vs. 17%) but a lower incidence of pulmonary toxicity (< 1% vs. 3%) than patients receiving ABVD. Neutropenia (54% vs. 39%) was the most frequently encountered SAE in both cohorts. Prophylaxis with granulocyte colony-stimulating factor (G-CSF) effectively decreased the rate of neutropenia and febrile neutropenia [51, 63].

BV plus etoposide, cyclophosphamide, doxorubicin, dacarbazine, and dexamethasone (BrECADD), a modified first-line treatment for advanced classical HL by incorporating BV was reported with an 88% CR rate and a more favorable toxicity profile (NCT01569204) [64]. This BrECADD regimen is currently being compared to the standard BEACOPP chemotherapy in a phase III randomized trial (HD21, NCT02661503).

BV has been studied in a few subtypes of non-Hodgkin lymphoma (NHL), including systemic ALCL, an aggressive CD30^+^ subtype of peripheral T cell lymphoma (PTCL). In a phase II multicenter trial (NCT00866047), 58 patients with systemic ALCL after failure of at least one prior multiagent chemotherapy regimen received intravenous BV 1.8 mg/kg every 3 weeks. Fifty (86%) patients achieved ORR, and 33 (57%) achieved CR [65]. For all enrolled patients, the estimated 5-year OS and PFS were 60% and 39% respectively. Among those who achieved CR, the 5-year OS and PFS were 79% and 57% respectively. Of the 50 patients with an objective response, the median duration of response (DOR) was 25.6 months [66]. In 2018, FDA approved BV in combination with cyclophosphamide, doxorubicin, and prednisone (CHAP) for the treatment of CD30-expressing PTCL including systemic ALCL, angioimmunoblastic T cell lymphoma, and PTCL not otherwise specified, based on the positive result of a randomized, double-blind phase III trial (ECHELON-2, NCT01777152). In this study, 452 PTCL patients were randomized to receive either CHAP or cyclophosphamide, doxorubicin, vincristine, and prednisone (CHOP). The patients in the CHAP group had longer PFS (48.2 months vs. 20.8 months), a higher ORR (83% vs. 72%), and CR rate (68% vs. 56%). Most common AEs of CHAP recipients were nausea (46%), peripheral neuropathy (45%), neutropenia (38%), and diarrhea (38%), comparable to those of CHOP recipients. Neutropenia (35%) was the most common SAE [67]. BV has also been investigated in cutaneous T cell lymphoma (CTCL). In a multicenter randomized phase III trial (ALCANZA, NCT01578499) that enrolled adult patients with CD30-positive mycosis fungoides or primary cutaneous ALCL who had received prior systemic therapy, patients receiving BV demonstrated more favorable outcome than those receiving conventional therapy (methotrexate or bexarotene): ORR lasting at least 4 months (56.3% vs. 12.5%), CR (16% vs 2%), and median PFS (17.2 vs. 3.5 months). A higher level of CD30 expression seemed to be associated with a better response to treatment with BV. There were mostly grade 1 to 2 AEs in BV-treated patients. Of note, the incidence of reported peripheral neuropathy (45%; grade ≥ 3: 5%) was much higher than that in patients receiving conventional therapy. Other common AEs included nausea (36%), diarrhea (29%), and fatigue (29%) [68].

In addition, several studies suggested that BV could provide additional treatment options for patients suffering from R/R B-cell NHL [69, 70]. In one phase II trial (NCT01421667) that enrolled 49 patients with heavily pretreated diffuse large B cell lymphoma (DLBCL), there was a 44% ORR with a 17% CR, and the DOR was 16.6 months [69].

### Inotuzumab ozogamicin (Besponsa®, CMC-544)

Inotuzumab ozogamicin (InO) is composed of a humanized anti-CD22 IgG4 mAb conjugated to calicheamicin via an acid-labile linker [71]. CD22 is a B cell-restricted transmembrane sialoglycoprotein, present on the surface of mature B cells, and thought to be involved in signal transduction, B cell activation and regulation [11, 71]. CD22 is also expressed by most B cell malignancies, including leukemic blasts in > 90% patients with B cell ALL, as well as chronic lymphocytic leukemia (CLL), NHL, and hairy cell leukemia (HCL) [72, 73]. Therefore, CD22 represents an important therapeutic target for ALL and other B cell malignancies.

InO has been approved by the US FDA in 2017 for the treatment of R/R CD22-positive B cell ALL [9, 10]. The approval was mainly based on a phase III, international, randomized trial (INO-VATE, NCT01564784) designed to compare single-agent InO with chemotherapy regimens as first or second salvage therapy for R/R ALL patients. Three hundred thwenty-six adult patients were randomized to receive either InO (*n* = 164) or standard of care (SoC, intensive chemotherapy; *n* = 162) [74]. The CR/CR with incomplete hematologic recovery (CRi) rate was significantly higher in patients in the InO arm (73.8%) compared to those in the SoC arm (30.9%). Both PFS and OS were longer with InO than SoC: the median PFS was 5.0 vs. 1.7 months, the median OS was 7.7 vs. 6.2 months, and the 2-year OS rate was 22.8% vs. 10.0%. Subset analyses revealed that remission rates remained consistent for patients with Philadelphia chromosome-positive (Ph+) or -negative (Ph−) ALL. Interestingly, more patients in the InO arm proceeded directly to AlloSCT after achieving CR/CRi (39.6% vs. 10.5%), suggesting InO as an effective bridging therapy to transplantation [75]. Besides, InO-treated patients reported a better quality of life than those receiving SoC [76]. Frequent AEs reported by InO recipients were neutropenia (36%), thrombocytopenia (29%), anemia (18%), febrile neutropenia (16%), nausea (15%), and pyrexia (11%), with lower incidences than SoC recipients [74]. However, there is a higher incidence of hepatotoxicity especially veno-occlusive disease (VOD) (14% vs. 2.1%), which is also the most common SAE found in InO recipients. Of note, among patients who proceeded to AlloSCT, 22% of InO recipients developed VOD after transplantation compared to only 3% of SoC recipients [77]. In view of this risk, expert opinions recommended that for patients planning to receive AlloSCT, InO administration should be limited to two cycles. Transplantation conditioning regimens containing two alkylating agents or any concomitant hepatotoxic drugs should be avoided [78]. Besides, patients receiving InO should also be monitored for prolonged QTc and tumor lysis syndrome. Interestingly, weekly low doses of InO has been revealed to result in less AEs than single-dose InO [79]. Currently, there is an ongoing trial evaluating the efficacy of weekly low-dose InO for R/R CD22-positive ALL (NCT03094611).

In addition to monotherapy, InO has been studied in combination with chemotherapy for R/R B cell ALL. InO was added to the treatment cycles of mini-hyper-CVD (miniHCVD), which was a modified hyper-CVAD regimen (hyper-fractionated cyclophosphamide, vincristine, doxorubicin, and dexamethasone) with no anthracycline, whereas cyclophosphamide and dexamethasone were given at 50% dose reduction, methotrexate at 75% dose reduction, and cytarabine at 0.5 mg/m^2^ for four doses [80]. InO was added on day 3 of each cycle with 1.3 mg/m^2^ in cycle 1 and 1 mg/m^2^ in subsequent cycles. A phase II trial (NCT01371630) enrolled 59 patients with R/R B cell ALL receiving InO-miniHCVD. The regimen showed a 78% ORR (59% CR) and a minimal residual disease (MRD) negativity of 82% among responders. The median OS and PFS were 11 and 8 months respectively, and the 1-year overall OS and PFS rates were 46% and 40% respectively. Subset analysis showed that the OS rate was much higher in patients who were treated in first salvage than those in subsequent salvages. Notably, 44% patients proceeded to subsequent ASCT suggesting InO + miniHCVD as an option for bridging therapy. The most common SAEs reported were thrombocytopenia (81%) and infections (73%). VOD still occurred in 15% of patients [81]. Even though InO + miniHCVD seemed to present with more favorable outcomes compared to the INO-VATE trial, further phase III studies are still warranted to ascertain the value of this combination therapy.

InO-miniHCVD has also been investigated as a frontline therapy in older patients (≥ 60 years) with newly diagnosed Ph− B cell ALL [82]. A single-arm phase II trial (NCT01371630) conducted in MD Anderson Cancer Center first reported that InO-miniHCVD demonstrated robust activity (ORR 98%, CR 85%, 3-year OS 56%, and 3-year PFS 49%). Frequently reported SAEs were thrombocytopenia (81%), infections occurred during induction (52%) or consolidation (69%), hyperglycemia (54%), and hepatic events including VOD (33%) [82]. In an attempt to further reduce toxicity and length of maintenance, blinatumomab (blina), a bispecific T cell-engaging antibody targeting both CD19 and CD3 [83–86], was added to the regimen. This new regimen, InO-miniHCVD with or without blina, achieved a 98% ORR (87% CR) and a 54% 3-year OS according to the interim result of an ongoing phase II trial (NCT03249870), comparable to the previous one [87]. A recently published retrospective propensity score analysis reported that older patients with newly diagnosed Ph− ALL who received InO-miniHCVD ± blina demonstrated better outcomes and lower risks than those who received hyper-CVAD (ORR 98% vs. 88%; 3-year OS 64% vs. 34%; early death 0% vs. 8%) [88]. Currently, there is another ongoing trial examining combination of InO with hyper-CVAD as a frontline therapy for ALL patients (NCT03488225).

The development of InO in B cell lymphoma has made relatively less progress. Single-agent InO was administered to 81 patients with R/R B cell NHL, predominantly follicular lymphoma (FL), who failed rituximab, rituximab plus chemotherapy, or radioimmunotherapy in a phase II trial (NCT00868608). The study reported a 67% ORR, a 31% CR, and a median PFS of 12.7 months. Bone marrow suppression was reported with most frequently thrombocytopenia (74%) and neutropenia (56%). Of note, 58% of patients with AEs discontinued the treatment [89]. InO combined with rituximab (R-InO) is thought to be a treatment option for patients with R/R B cell NHL who are not candidates for high-dose chemotherapy. One phase I/II trial (NCT00299494) reported that the R-InO regimen at MTD (1.8 mg/m^2^ InO plus 375 mg/m^2^ rituximab) yielded an 87% ORR for R/R FL and 74% ORR for R/R DLBCL. R-InO demonstrated a similar toxicity profile to the single-agent InO [90]. A separate phase II trial (NCT00867087) reported only 29% ORR after 3 cycles of R-InO for patients with R/R DLBCL [91]. However, a recent randomized phase III trial (NCT01232556) revealed that the outcome of the R-InO recipients appeared not to be superior to those receiving rituximab plus chemotherapy of bendamustine or gemcitabine in terms of ORR (41% vs. 44%), OS (35% vs. 37%), and PFS (19% vs. 17%) [92]. Several clinical trials are exploring different new regimens. InO at 0.8 mg/m^2^ combined with full dose of rituximab, cyclophosphamide, vincristine, and prednisolone (R-CVP) was reported safe and effective for CD22^+^ R/R B cell NHL in a phase I trial (NCT01055496). The ORR was 84% with 24% CR. Subset analysis showed that ORR was 100% for patients with indolent lymphoma and 57% for those with aggressive histology. The toxicity profile was also similar to InO monotherapy [93]. Currently, InO + R-CVP is being evaluated in patients with DLBCL not suitable for anthracycline-based chemotherapy in a phase II trial (NCT01679119). InO at 0.8 mg/m^2^ plus full dose rituximab, gemcitabine, dexamethasone, and cisplatin (R-GDP) is a regimen proposed by another phase I trial (NCT01055496) for patients with R/R CD22^+^ B cell NHL. The preliminary result was less encouraging (53% ORR; 20% CR) [94].

### Moxetumomab pasudotox (Lumoxiti®, CAT-8015)

Moxetumomab pasudotox (MP) is the second anti-CD22 ADC that comes into clinical practice. It has the Fv fragment of a recombinant murine mAb with higher affinity for CD22 than the parent compound [95]. The fragment is genetically fused to a truncated form of *Pseudomonas aeruginosa* exotoxin (PE38) [96]. MP has been approved by the US FDA in September 2018 for the treatment of adult patients with R/R hairy cell leukemia (HCL) who have received at least two prior systemic therapies, including two courses of a purine analog or one course of rituximab or a BRAF inhibitor following a single course of a purine analog. The approval was mainly based on a pivotal, multicenter, open-label, single-arm trial (NCT01829711) that enrolled 80 patients who received 40 μg/kg MP intravenously on days 1, 3, and 5 every 28 days for a maximum of 6 cycles. The treatment led to a 75% ORR with 41% CR. Eighty-five percent of CR patients achieved MRD negativity. PFS was not yet reached at a median follow-up of 16.7 months. The median DOR for the MRD-positive patients was 5.9 months and has not been reached for MRD-negative patients [97]. Elimination of MRD has been shown to be associated with prolonged CR duration [98]. The most common treatment-emerging AEs (TEAE) were peripheral edema (39%), nausea (35%), fatigue (34%), and headache (33%). Serious AEs included hemolytic uremic syndrome (HUS) (8%) and capillary leak syndrome (CLS) (5%). Although four of HUS and two of CLS patients ended up with discontinuation of therapy, most of the above AEs were manageable with dose reduction and supportive care [97, 99]. It was noted that antidrug titers increased after repeated administration of the ADC [99]. Currently, a phase I trial (NCT03805932) is looking at whether the combination of MP and rituximab is safe and effective for R/R HCL. The development of MP for precursor cell lymphoblastic leukemia/lymphoma, NHL, and CLL has been discontinued [99].

### Polatuzumab vedotin (Polivy®, DCDTS4501A)

CD79b is a component of the B cell receptor (BCR) complex [100]. It is a promising therapeutic target because of its restricted expression on mature B cells and B cell malignancies [101]. Polatuzumab vedotin (Pola) is an anti-CD79b mAb conjugated to MMAE via a protease-cleavable linker similar to the structure of anti-CD22 ADC, pinatuzumab vedotin (Pina). Phase I trials demonstrated that single-agent Pola at a recommended dose of 2.4 mg/kg was effective against NHL but not for CLL [102]. Both pola and pina were often studied in combination with rituximab. The ROMULUS trial showed that irrespective of CD79b expression, R-Pola was associated with a 54% ORR (21% CR), and a median PFS of 5.6 months for DLBCL patients, which was comparable to those from R-Pina; but for FL patients, R-Pola was associated with a 70% ORR (45% CR), and a median PFS of 15.3 months, much more effective than R-Pina. R-Pola shared a similar profile of AEs with R-Pina, but less incidence of SAEs [103]. Therefore, the overall risk-benefit ratio favored Pola for further investigation in B cell NHL.

In an ongoing randomized phase II trial (NCT02257567), Pola was added to the regimen of bendamustine plus rituximab (BR) or obinutuzumab (BO) for patients with R/R DLBCL or R/R FL ineligible for autologous stem cell transplantation (ASCT). The interim result showed that the addition of Pola significantly improve ORR (45% vs. 18%), CR (40% vs. 18%), median OS (12.4 vs. 4.7 months), and PFS (7.6 vs. 2.0 months) [104]. For the R/R DLBCL patients in this randomized phase II trial, 40 patients received Pola-BR, and 40 patients were randomized to BR only arm. In the Pola-BR arm, 25 (63%) patients achieved a PR/CR, versus 25% in the BR arm. The DOR was also longer in the pola-BR arm. Pola has been approved by FDA in June 2019 for R/R DLBCL who have failed at least two prior therapy and are not eligible for ASCT (Table 1). Pola is infused at 1.8 mg/kg over 90 min in combination with BR for the first cycle. Subsequent infusions may be administered over 30 min if the previous infusion is tolerated. The Pola-BR regimen is given every 21 days for a total of 6 cycles. One phase I/II trial (NCT01992653) reported that Pola plus obinutuzumab, cyclophosphamide, doxorubicin, and prednisone (Pola-G-CHP) yielded a 91% ORR (81% CR) with manageable toxicity profile. Common SAEs were neutropenia (38%) and febrile neutropenia (33%) [105]. Another phase I/II trial (NCT01992653) investigated the combination of Pola with rituximab-cyclophosphamide, doxorubicin, and prednisone (Pola-R-CHP). An encouraging response with 91% ORR (78% CR) was also achieved. Most common SAEs were neutropenia (27%) and febrile neutropenia (11%) [106]. A multicenter, randomized, double-blind, placebo-controlled phase III trial (POLARIX, NCT03274492) is currently ongoing to compare Pola-R-CHP to rituximab-cyclophosphamide, doxorubicin, vincristine, and prednisone (R-CHOP) as the first-line treatment for patients with newly diagnosed DLBCL.

## ADCs under clinical development for lymphoid malignancies

### Anti-CD19 ADCs

CD19 is a transmembrane glycoprotein that is essential in modulating both B cell receptor-dependent and independent signaling. It is ubiquitously expressed in the B lymphocyte lineage, from the earliest B lineage committed cells, and continuing through pre-B and mature B cell stage. CD19 is present in the majority of B cell malignancies. CD19 is therefore a genuine biomarker for normal and malignant B cells, making it an ideal therapeutic target [107].

Coltuximab ravtansine (SAR3419) is a humanized anti-CD19 IgG1 mAb conjugated to DM4 via a cleavable disulfide linker [108] (Table 2). In a phase II trial (NCT01472887), SAR3419 was administered to patients with R/R DLBCL previously treated with rituximab-containing regimen. The dose used in this study was 55 mg/m^2^ weekly for 4 weeks, then every 2 weeks for the next four doses. The study achieved a 43.9% ORR (14.6% CR), and a median OS and PFS of 9.2 and 4.4 months respectively. In contrast to limited efficacy, the safety profile was favorable. Common AEs were mainly gastrointestinal disorders such as diarrhea and nausea (43%), asthenia (30%), and ocular toxicity (25%). Most common SAEs were hepatotoxicity (3%) and abdominal pain (3%) [109]. Another phase II trial combined SAR3419 with rituximab for R/R DLBCL, but the trial resulted in a less favorable clinical response compared to the previous single-agent trial [119]. SAR3419 has also been evaluated in patients with R/R ALL in a phase II trial (NCT01440179), but ORR was only 25.5%. This trial was terminated [120].

**Table 2:** Antibody-drug conjugates in clinical trials for lymphoid malignancies

ADC names	Target	Linker	Payload	Indications	Major responses	Status [reference]
Brentuximab vedotin (Adcetrix®)	CD30	Protease-cleavable (Val-Cit)	MMAE	R/R HL	ORR 75%, CR 34%; OS 40.5 months, PFS 9.3 months; 5-year OS 41%, PFS 22%;	Approved [54]
				Frontline stage III & IV HL (+ AVD)	ORR 86%, CR 73%, 2-year PFS 81%	Approved [51]
				Post-ASCT consolidation for HL	PFS 42.9 months, 5-year PFS 59%	Approved [60, 61]
				R/R systemic ALCL	ORR 86%, CR 57%, 5-year OS 79%, PFS 57%, DOR 25.6 months	Approved [65, 66]
				R/R PTCL (+ CHP)	ORR 83%, CR 68%, PFS 48.2 months	Approved [67]
				R/R CTCL	ORR 56.3%, CR 16%, PFS 17.2 months	Approved [68]
Inotuzumab ozogamicin (Besponsa®)	CD22	Acid-labile (hydrazone)	Calicheamicin	R/R B-cell ALL,	CR/CRi 73.8%, OS 7.7 mo, PFS 5 months	Approved [74, 75]
				Frontline Ph- B-cell ALL (+ miniHCVD)	ORR 98%, CR 85%, 3-year OS 56%, PFS 49%	Phase II [82]
				R/R B-cell NHL	ORR 67%, CR 31%, PFS 12.7 months	Phase II [89]
Moxetumomab pasudotox (Lumoxiti®)	CD22	Disulfide bond	PE38	R/R HCL	ORR 75%, CR 41%, PFS not reached at 16.7-month follow up	Approved [97, 98]
Polatuzumab vedotin (DCDTS4501A)	CD79b	Protease-cleavable (Val-Cit)	MMAE	R/R DLBCL (+ BR)	ORR 63%, PFS 7.6 months, OS 12.4 months	Approved [103]
				Newly diagnosed DLBCL (+ G-CHP or + R-CHP)	G-CHP: ORR 91%, CR 81%; R-CHP: ORR 91%, CR 78%;	Phase III [105, 106]
Coltuximab ravtansine (SAR3419)	CD19	Disulfide bond (SPDB)	DM4	R/R DLBCL	ORR 43.9%, CR 14.6%, OS 9.2 months, PFS 4.4 months	Phase II [109]
Denintuzumab mafodotin (SGN-CD19A)	CD19	Non-cleavable (MC)	MMAF	R/R B-cell ALL	ORR 35%, CR 19%, DOR 27 weeks	Phase I [110]
				R/R DLBCL	ORR 33%, CR 22%, DOR 40 weeks	Phase I [111]
Loncastuximab Tesirine (ADCT-402)	CD19	Protease-cleavable (Val-Ala)	PBD dimer	R/R B-cell ALL	CR 8.7%	Phase I [112]
				R/R NHL	DLBCL: ORR 40.2%, CR 22% MCL: ORR 46.7%, CR 26.7%; FL: ORR 80%, CR 53.3%	Phase I [113, 114]
Pinatuzumb vedotin (DCDT2980S)	CD22	Protease-cleavable (Val-Cit)	MMAE	R/R B-cell NHL (+ Rituximab)	DLBCL: ORR 60%, CR 26%, PFS 5.4 months; FL: ORR 62%, CR 5%, PFS 12.7 months	Phase II [103]
Camidanlumab tesirine (ADCT-301)	CD25	Protease-cleavable (Val-Ala)	PBD dimer	R/R HL	ORR 80.8%, CR 50%, DOR 7.7 months, PFS 6.7 months	Phase I [115]
				R/R B and T cell NHL	B: ORR 31.3%, CR 18.8%; T: ORR 50%, CR 0	Phase I [116]
Naratuximab emtansine (IMGN529)	CD37	Non-cleavable (SMCC)	DM1	R/R B-cell NHL	ORR 13%, CR 2.6%	Phase I [117]
AGS67E	CD37	Protease-cleavable (Val-Cit)	MMAE	R/R B and T cell NHL	ORR 22%, CR 14%	Phase I [118]

*ADC* antibody-drug conjugate, *ALCL* anaplastic large cell lymphoma, *ALL* acute lymphoblastic leukemia, *CTCL* cutaneous T cell lymphoma, *HL* Hodgkin lymphoma, *NHL* non-Hodgkin lymphoma, *R*/*R* relapsed/refractory, *MM* multiple myeloma, *CR* complete remission, *DLBCL* diffuse large cell lymphoma, *DOR* duration of response, *FL* follicular lymphoma, *MCL* mantle cell lymphoma, *ORR* overall response rate, *OS* overall survival, *PFS* progression-free survival, *PTCL* peripheral T cell lymphoma

Denintuzumab mafodotin (SGN-CD19A) is a humanized anti-CD19 IgG1 mAb conjugated to MMAF via a non-cleavable MC linker. A phase I trial (NCT01786096) of SGN-CD19A showed a 35% ORR (19% CR) and a median DOR of 27 weeks in patients with R/R B cell ALL. Most frequently reported AEs were pyrexia (54%), nausea (52%), fatigue (51%), headache (44%), chills (38%), vomiting (37%), and blurry vision (35%) [110]. A separate phase I trial (NCT01786135) in patients with R/R DLBCL revealed a 33% ORR (22% CR) and a median DOR of 40 weeks. Of note, ocular toxicity, such as blurry vision (65%), dry eye (52%), and keratopathy (35%), was prominent [111]. Two phase II trials were evaluating the combination of SGN-CD19A and other regimens (NCT02855359: R-CHP or R-CHOP; NCT02592876: RICE [rituximab, ifosfamide, carboplatin, and etoposide]) in DLBCL, but were currently on hold.

Loncastuximab tesirine (ADCT-402) is a humanized anti-CD19 IgG1 mAb conjugated through a protease-cleavable Val-Ala linker to a PDB dimer, a DNA crosslinking agent. ADCT-402 was investigated in a phase I trial (NCT02669264) of adult patients with R/R B-cell ALL. The interim result showed that only two out of 23 patients (8.7%) achieved CR with MRD negativity. The most frequently reported TEAEs were nausea (30%), fatigue (26%), and febrile neutropenia (22%), with febrile neutropenia being the most common SAE [112]. Another ongoing phase I trial (NCT02669017) of ADCT-402 in R/R B cell NHL recently reported its interim result. ADCT-402 yielded a 40.2% ORR (22% CR), with a median DOR and PFS of 4.17 and 2.79 months respectively at doses > 120 μg/kg in a subpopulation of 132 evaluable patients with R/R DLBCL. Most common SAEs were neutropenia (42%), thrombocytopenia (27.3%), anemia (11.7%), and increased gamma-glutamyltransferase (GGT) (8.8%) [113]. Among the 132 patients, 15 evaluable patients with mantle cell lymphoma (MCL) treated with ADCT-402 achieved a 46.7% ORR (26.7% CR) and a median DOR and PFS of 5.3 and 4.8 months respectively; while for the 15 evaluable patients with FL, ADCT-402 therapy achieved an 80% ORR (53.3% CR), the median DOR and PFS have not been reached at the 7.56-months follow-up. The most common SAEs were elevated GGT (26.7%), neutropenia (20%), and anemia (13.3%) [114]. Another phase II trial (NCT03589469) in patients with R/R DLBCL is ongoing to evaluate the efficacy and safety of single agent ADCT-402. In addition, there are phase II trials looking at the combination therapy of ADCT-402 and ibrutinib (NCT03684694), ADCT-402 and durvalumab, a PD-L1 blocker (NCT03685344) for R/R DLBCL, FL, or MCL.

### Anti-CD22 ADCs

Pinatuzumab vedotin (Pina, DCDT2980S) is another anti-CD22 ADC currently under clinical development. Pina is a humanized anti-CD22 IgG1 mAb conjugated to MMAE via a protease-cleavable linker [121]. Pina has been revealed effective with or without rituximab for R/R DLBCL and R/R indolent NHL by a phase I trial (NCT01209130), but little effect was seen in CLL [122]. A multicenter phase II trial (ROMULUS, NCT01691898) randomized 81 patients with R/R DLBCL and 42 patients with R/R FL to receive rituximab plus either Pina (R-Pina) or polatuzumab vedotin (anti-CD79b ADC, discussed above). The recommended dosage was Pina or Pola 2.4 mg/kg with rituximab 375 mg/m^2^ every 21 days up to 1 year or until disease progression or severe toxicity. The regimen of R-Pina generated a 60% ORR (26% CR) and a median PFS of 5.4 months for the DLBCL cohort, whereas a 62% ORR (5% CR) and a median PFS of 12.7 months were seen for the FL cohort. Neutropenia (29%) was the most common SAE. Peripheral neuropathy was a major AE that led to discontinuation of treatment in 21% of patients with DLBCL and 48% of patients with FL [103].

### Anti-CD25 ADCs

CD25 is the α subunit of IL-2 receptor (IL-2Rα) involved in the signal transduction for the growth and survival of immune cells. CD25 overexpression has been found in multiple hematologic malignancies including both HL and NHL, as well as ALL, B cell CLL, HCL, and adult T cell leukemia (ATL) [123]. Camidanlumab tesirine (ADCT-301) is the first ADC developed targeting CD25. ADCT-301 consists of a humanized IgG1 mAb (HuMax®-TAC) conjugated to a PBD dimer warhead via a protease-cleavable linker. ADCT-301 is being investigated in R/R HL and NHL in a phase I trial (NCT02432235). Among 26 patients with R/R HL who failed prior BV treatment, therapy with ADCT-301 at a dose of 45 μg/kg achieved an 80.8% ORR (50% CR). Median DOR and PFS were 7.7 and 6.7 months, respectively. Major SAEs included GGT elevation (16.7%) and maculopapular rash (13.3%) [115]. The same trial also enrolled 16 patients with B cell NHL and ten patients with T cell NHL who failed a median of three prior therapies. These patients were treated with ADCT-301 at a dose range of 60–150 μg/kg. The B cell NHL cohort demonstrated a 31.3% ORR (18.8% CR), while the T cell NHL cohort demonstrated a 50% ORR (all PR) [116]. ADCT-301 has also been evaluated in patients with ALL and acute myeloid leukemia (AML) in another phase I trial (NCT02588092). Even though the safety profile was acceptable, no response has been reported to date [124].

### Anti-CD37 ADCs

CD37 is a member of transmembrane tetraspanin protein family. Similar to CD19, CD37 is almost exclusively expressed on B cells, but absent from hematopoietic stem cells and plasma cells. It plays a role in signal transduction and immune cell interactions that are important for B cell activation and survival [125]. CD37 is also highly expressed on malignant B cells including most histological subtypes of NHL [126]. Two ADCs targeting CD37, IMGN529 and AGS67E, are currently under clinical development.

Naratuximab emtansine (IMGN529) is a humanized anti-CD37 IgG1 antibody conjugated to the maytansinoid DM1 via a nonreducible thioether linker, succinimidyl-4-(*N*-maleimidomethyl)-cyclohexane-1-carboxylate (SMCC) [127]. Of note, IMGN529 exhibited significant intrinsic ADCC activity against targeted cells [128]. A phase I trial (NCT01534715) evaluated the safety and efficacy of IMGN529 monotherapy with escalating doses in R/R B cell NHL. The MTD was 1.4 mg/kg every 3 weeks. Only five out of 39 (13%) evaluable patients achieved ORR (one CR), four of whom were patients with DLBCL. The most common AEs were neutropenia (37%), thrombocytopenia (37%), and pyrexia (37%) [117]. Rituximab has been reported to potentiate the antitumor effect of IMGN529 in multiple xenograft models [128]. The combination of IMGN529 and rituximab for patients with R/R NHL is being explored in a phase II trial (NCT02564744).

AGS67E is a fully human anti-CD37 IgG2 mAb conjugated to MMAE via a protease-cleavable linker. Preclinical study discovered that AGS67E was able to alter cell cycle and induce apoptosis in vitro and in xenograft models of NHL [129]. AGS67E demonstrated some effects in a phase I trial (NCT02175433) in 50 patients with R/R B and T cell NHL. Eleven (22%) patients experienced ORR, and seven (14%) patients achieved CR, four of whom had DLBCL. Peripheral neuropathy (16%) and neutropenia (8%) were the major AEs [118].

### Anti-CD70 ADCs

CD70, also known as CD27L, is a member of TNF receptor superfamily. The interaction between CD70 and CD27 is critical for B cell activation, T helper 1 (Th1)/Th2 switching, and cell differentiation. CD70 is highly expressed on several malignancies including NHL and renal cell carcinoma (RCC) [130]. Three anti-CD70 ADCs have been investigated in phase I trials in patients with CD70-positive R/R B-cell NHL and metastatic RCC.

SGN-CD70A is a humanized anti-CD70 IgG1 mAb conjugated to a PBD dimer via a protease-cleavable linker. Severe (grade ≥ 3) early-onset thrombocytopenia was reported in 75% of patients with DLBCL and MCL treated by SGN-CD70A and led to treatment termination (NCT02216890) [131]. MDX-1203 (BMS-936561) consists of a fully human anti-CD70 IgG1 mAb conjugated to a duocarmycin derivative through a protease-cleavable linker. In a dose-escalation phase I trial (NCT00944905), dose-limiting toxicity of grade 3 hypersensitivity (13%) and delayed toxicities featured by facial edema and pleural/pericardial effusions (38%) were recorded. Eighteen of 26 patients (69%) maintained stable diseases. There was no dose-response correlation [132]. Vorsetuzumab mafodotin (SGN-75) is a humanized anti-CD70 IgG1 mAb conjugated to MMAF via a non-cleavable linker. However, the development of SGN-75 for NHL was discontinued due to idiopathic thrombocytopenia purpura in two out of 19 NHL patients in a phase I trial (NCT01015911) [133]. It appears that these three anti-CD70 ADCs have major concerns over the toxicity profiles which led to termination of further clinical development.

## ADCs under clinical development for multiple myeloma

### Anti-CD56 ADC

CD56, also called neural cell adhesion molecule 1 (NCAM1), is a marker for cells of neuroendocrine origin, as well as natural killer cells and a subset of T cells. It is also expressed on 75% of malignant plasma cells but less than 15% of normal plasma cells. These features make it an attractive therapeutic target for multiple myeloma (MM) [134]. Lorvotuzumab mertansine (IMGN901) is a humanized anti-CD56 IgG1 mAb conjugated to DM1 via a stable disulfide linker. IMGN901 retains the ADCC activity [135] (Table 3). Single-agent IMGN901 has been evaluated in patients with CD56-positive MM and small-cell lung cancer. In a dose-escalating phase I trial (NCT00346255) that enrolled 37 patients with R/R MM, there was only a 5.7% ORR with no CR, but 42.9% of patients remained in stable disease for an average duration of 15.5 months. The AE profile was acceptable, with the MTD at 112 mg/m^2^ [136]. IMGN901 is being studied in combination with lenalidomide and dexamethasone in a phase I trial (NCT00991562) in patients with R/R MM. Among 32 patients, the ORR was 59% with 1 CR [140].

**Table 3:** Antibody-drug conjugates in clinical trials for multiple myeloma

ADC names	Target	Linker	Payload	Disease	Major responses	Status [reference]
Lorvotuzumab mertansine (IMGN901)	CD56	Disulfide bond (SPP)	DM1	R/R MM	ORR 5.7%, CR 0; 42.9% stable disease for 15.5 mo	Phase I [136]
Milatuzumab doxorubicin (hLL1-DOX)	CD74	Acid-labile (hydrazine)	Doxorubicin	R/R MM	Data not reported	Phase I [137]
Indatuximab ravtansine (BT062)	CD138	Disulfide bond (SPDB)	DM4	R/R MM	ORR 5.9%, CR 0, stable disease 61.8%, OS 26.7 mo, PFS 3 mo	Phase I/II [138]
GSK2857916	CD269 (BCMA)	Non-cleavable (MC)	MMAF	R/R MM	ORR 60%, CR 14%, PFS 12 mo, DOR 14.3 mo	Phase I [139]

*ADC* antibody-drug conjugate, *BCMA* B cell maturation antigen, *R*/*R* relapsed /refractory, *MM* multiple myeloma, *CR* complete remission, *DOR* duration of response, *ORR* overall response rate, *OS* overall survival, *PFS* progression free survival, *Mo* month

### Anti-CD74 ADC

CD74 is a type II transmembrane glycoprotein involved in major histocompatibility complex class II antigen presentation, B cell maturation, and T cell response. CD74 is expressed in more than 90% of B cell malignancies. It becomes an attractive therapeutic target because of its rapid internalization and recycling [141]. Milatuzumab doxorubicin (hLL1-DOX) is a humanized CD74 mAb conjugated to doxorubicin via an acid-labile hydrazone linker [137]. Unconjugated milatuzumab effectively maintained five out of 19 patients with MM (26%) in stable disease for more than 3 months in a phase I trial (NCT00421525) [142]. hLL1-DOX has been administered to 17 patients with R/R MM in a phase I trial (NCT01101594) which has not reported any updates so far.

### Anti-CD138 ADC

CD138, also known as syndecan-1, is a transmembrane protein receptor for extracellular matrix involved in cell-cell adhesion. It is upregulated on malignant plasma cells, as well as various epithelial neoplasms [143]. Indatuximab ravtansine (BT062) is a chimeric B-B4, an afucosylated IgG4 mAb specific for CD138, conjugated to maytansinoid DM4 via a SPDB disulfide cleavable linker. Preclinical studies have demonstrated the efficacy of BT062 against the growth of MM cells in vivo [144]. In a phase I trial (NCT01001442) conducted in 35 patients with R/R MM who have failed previous immunomodulatory drugs and proteasome inhibitor therapies, BT062 was administered at the MTD of 140 mg/m^2^ on days 1, 8, and 15 of each 28-day cycle. The therapy resulted in a 5.9% ORR with no CR. Then, 61.8% of patients achieved stable diseases. The median OS and PFS were 26.7 and 3 months respectively. Eight-eight percent reported AEs were grade 1–2, with the most common ones being fatigue (47.7%) and diarrhea (43.2%) [138]. The same regimen was investigated in combination with dexamethasone (20–40 mg on days 1, 8, 15, and 22) and lenalidomide (25 mg, daily on days 1–21) in another phase I/IIa trial (NCT01638936). Among 43 evaluable patients who completed at least 2 cycles of treatment, 33 (77%) achieved ORR (no CR) with a median DOR of 21.0 months. Frequently reported AEs were fatigue, diarrhea, and nausea [145].

### Anti-CD269 (BCMA) ADC

CD269, also known as B cell maturation antigen (BCMA), is a transmembrane receptor required for B cell maturation. It is universally expressed on malignant plasma cells. Increased plasma level of BCMA is associated with a worse prognosis of MM [146, 147]. GSK2857916 is a humanized anti-BCMA IgG1 mAb conjugated to MMAF via a MC non-cleavable linker. Additionally, GSK2857916 is able to induce enhanced activity of ADCC against MM cells [148]. A multicenter phase I trial (NCT02064387) enrolled 35 adult patients with R/R MM after ASCT, alkylators, proteasome inhibitors, and immunomodulators. At a recommended dose of 3.4 mg/kg, 21 (60%) patients showed ORR with 5 (14%) achieving CR in a recent update. The median PFS and DOR were 12 and 14.3 months respectively. There were mostly grade 1-2 AEs, with thrombocytopenia (35%) being the most common SAE. Corneal events such as blurry vision (52%), dry eyes (37%), and photophobia (29%) were reported [139]. Several phase II trials are currently recruiting to investigate combination regimens incorporating GSK2857916 for patients with R/R MM. One study is exploring GSK2857916 plus pembrolizumab (NCT03848845). Another ongoing study is looking at GSK2857916 administered in combination with dexamethasone plus either lenalidomide or bortezomib (NCT03544281).

### Future perspectives

Careful clinical trials are needed for ADCs since several clinical trials of new ADCs have been terminated due to minimal efficacy and unacceptable toxicity. Efforts are being made on identifying better target antigens exclusively expressed on tumor cells to increase the specificity for targeted killing; exploring more potent payloads with high penetration ability and better bystander effect to enhance the antitumor activity; and optimizing the linker and conjugation technology to achieve a homogeneous DAR to enhance drug stability and minimize off-target toxicity. In addition, it is important to discover payloads that can avoid organ-specific toxicity, such as ozogamicin-related hepatic VOD.

A variety of antigen targets used for ADCs, such as CD19, CD22, and BCMA, are also being explored for engineering bispecific antibodies (BiTE) and chimeric antigen receptors (CAR) [149–153]. A CD 19 BiTE, blinatumomab, and two CAR T cell products have been approved for acute lymphoblastic leukemia [154, 155]. It remains to be determined whether any particular target (CD19, CD22, or BCMA) or form of targeted agents (ADC, BiTE, or CAR T) offers better therapeutic index. It is equally intriguing whether these immunotargeted agents can be combined concurrently or consequentially.

## Conclusion

Proper selections of antigen targets, linkers, and payloads are critical in ADC designs. Robust antitumor activity and favorable toxicity profiles of ADCs have made them an important modality of cancer therapy. ADCs in combination with chemotherapy regimens and immune checkpoint inhibitors are in clinical trials to further improve the treatment of lymphoid malignancies as well as multiple myeloma.

## Data Availability

The material supporting the conclusion of this review has been included within the article.

## Abbreviations

ADCC: Antibody-dependent cytotoxicity
Ala: Alanine
Cit: Citrulline
CDC: Complement-dependent cytotoxicity
DLT: Dose-limiting toxicity
DM1: N20-deacetyl-N20-(3-mercapto-1-oxopropyl)maytansine
DM4: N20-deacetyl-N20-(4-mercapto-4-methyl-1-oxopentyl)maytansine
MC: Maleimidocaproyl
MMAE: Monomethyl auristatin E
MMAF: Monomethyl auristatin F
PBD: Pyrrolobenzodiazepines
PE38: Pseudomonas aeruginosa exotoxin
SMCC: Succinimidyl-4-(*N*-maleimidomethyl)cyclohexane-1-carboxylate
SPDB: *N*-Succinimidyl-4-(2-pyridylthio)butanoate
SPP: *N*-Succinimidyl-4-(2-pyridyldithio)pentanoate
Val: Valine
