# Correction to: Antibody-drug conjugates in clinical trials for lymphoid malignancies and multiple myeloma

**DOI:** 10.1186/s13045-020-00902-5

**Published:** 2020-06-04

**Authors:** Bo Yu, Delong Liu

**Affiliations:** 1 Department of Medicine, Lincoln Medical Center, Bronx, NY USA; 2 Department of Oncology, The First affiliated Hospital of Zhengzhou University, Zhengzhou, China; 3 Department of Medicine, New York Medical College and Westchester Medical Center, Valhalla, NY USA

**Correction to: J Hematol Oncol (2019) 12:94**

**https://doi.org/10.1186/s13045-019-0786-6**

Drs Meghdad Abdollahpour-Alitappeh & Majid Lotfinia and their colleagues, Sajad Yaghoubi, Aliyar Pirouzi, Mohammad Hossein Karimi, Tohid Gharibi, Motahare Mahi-Birjand, Shiva Mohammadi, Koushan Sineh Sepehr, Nader Bagheri from Iran have written to the Journal of Hematology & Oncology regarding the article on antibody-drug conjugates (ADC) [1]. They raised the question that moxetumomab pasudotox (MS) is not an ADC. We appreciate their attention to the publication. We wish to make correction and clarification on this question. First of all, they are correct in pointing out that MS is not a conventional ADC which is usually an antibody conjugated to a cytotoxic chemical payload [2]. MS is an antibody conjugated to a cytotoxic bacterial toxin payload, which is commonly referred to as immunotoxin [3]. Considering the scope and topic of the contents we summarized on the clinical use of ADCs for lymphoma and multiple myeloma, we believe it may be better to keep the section in the article, but with the specific correction that MS is not a conventional ADC, rather an antibody-toxin conjugate, an immunotoxin.
